# Sorting Rare ALS Genetic Variants by Targeted Re-Sequencing Panel in Italian Patients: *OPTN*, *VCP*, and *SQSTM1* Variants Account for 3% of Rare Genetic Forms

**DOI:** 10.3390/jcm9020412

**Published:** 2020-02-03

**Authors:** Viviana Pensato, Stefania Magri, Eleonora Dalla Bella, Pierpaola Tannorella, Enrica Bersano, Gianni Sorarù, Marta Gatti, Nicola Ticozzi, Franco Taroni, Giuseppe Lauria, Caterina Mariotti, Cinzia Gellera

**Affiliations:** 1 Unit of Medical Genetics and Neurogenetics, Fondazione IRCCS Istituto Neurologico Carlo Besta, 20133 Milan, Italy; viviana.pensato@istituto-besta.it (V.P.); stefania.magri@istituto-besta.it (S.M.); pierpaola.tannorella@istituto-besta.it (P.T.); marta.gatti@istituto-besta.it (M.G.); franco.taroni@istituto-besta.it (F.T.); cinzia.gellera@istituto-besta.it (C.G.); 2 3rd Neurology Unit, Motor Neuron Diseases Centre, Fondazione IRCCS Istituto Neurologico Carlo Besta, 20133 Milan, Italyenrica.bersano@istituto-besta.it (E.B.); giuseppe.lauriapinter@istituto-besta.it (G.L.); 3 Department of Neuroscience, University of Padova, 35122 Padova, Italy; gianni.soraru@unipd.it; 4 Department of Neurology and Laboratory of Neuroscience, IRCCS Istituto Auxologico Italiano, 20149 Milan, Italy; n.ticozzi@auxologico.it; 5 Department of Pathophysiology and Transplantation, ‘Dino Ferrari’ Center, Università degli Studi di Milano, 20122 Milan, Italy; 6 Department of Biomedical and Clinical Sciences “Luigi Sacco”, University of Milan, 20157 Milan, Italy

**Keywords:** amyotrophic lateral sclerosis, next generation sequencing, gene panel, genetic heterogeneity, mutation screening

## Abstract

Amyotrophic lateral sclerosis (ALS) is an adult-onset progressive neurodegenerative disease due to motor neuron loss variably associated with frontotemporal dementia (FTD). Next generation sequencing technology revealed an increasing number of rare and novel genetic variants and interpretation of their pathogenicity represents a major challange in the diagnosis of ALS. We selected 213 consecutive patients with sporadic or familial (16%) ALS, tested negative for *SOD1*, *FUS*, *TARDBP*, and *C9orf72* mutations. To reveal rare forms of genetic ALS, we performed a comprehensive multi-gene panel screening including 46 genes associated with ALS, hereditary motor neuronopathies, spastic paraplegia, and FTD. Our study allowed the identification of pathogenic or likely pathogenic variants in 4.2% of patients. The genes with the highest percentage of pathogenic variants were *OPTN* (1%), VCP (1%) *SQSTM1(1%)*, *SETX (0.4%)*, *FIG4 (0.4%)*, and *GARS1 (0.4%*) genes. We also found 49 novel or rare gene variants of unknown significance in 30 patients (14%), 44 unlikely pathogenic variants (39%), and 48 variants in ALS susceptibility genes. The results of our study suggest the screening of *OPTN*, *VCP*, and *SQSTM1* genes in routine diagnostic investigations for both sporadic and familial cases, and confirm the importance of diagnosis and couselling for patients and their relative family members.

## 1. Introduction

Amyotrophic lateral sclerosis (ALS) is a severe neurodegenerative multisystem disorder characterized by loss of spinal, bulbar, and cortical motoneurons, leading to progressive and generalized paralysis. Death occurs within 3 to 5 years of onset, generally from respiratory complications [1]. The disease course may be variable and clinical presentation may include cognitive dysfunctions and behavioral changes resulting in the diagnosis of frontotemporal dementia [2]. ALS is an orphan disease with an incidence of 1–2 individuals per 100,000 each year in most countries, and a prevalence of about 5 cases per 100,000. The majority of the patients are sporadic (SALS), but in 10–20% of cases, the disease has a familial recurrence (FALS) implying a causative inheritable defect [3]. The absence of a suggestive family history, however, does not exclude a genetic cause for ALS, especially in small sized families and considering incomplete penetrance. The percentage of SALS caused by genetic variants ranges from 11% to 28% in populations of European ancestry [4,5].

Historically, the genetic studies in ALS began with the identification of missense mutations in *SOD1* (superoxide dismutase 1) gene in several families displaying an autosomal dominant transmission of the disease [6]. Clinical-genetic investigations demonstrated that *SOD1* is responsible for 20% of FALS and 3% of SALS, supporting the hypotesis of genetic heterogeneity in the disease. 

Over the last decade, the availability of next-generation high-throughput sequencing (NGS) revealed an increasing number of new ALS-associated genes, including causative genes, genetic modulating factors for symptom severity and/or progression, and susceptibility variants possibly increasing the risk of developing the disease [7,8].

The most frequent genetic cause of ALS was found to be a large hexanucleotide repeat expansion in an intronic region of the *C9orf72* gene [9,10]. This molecular defect accounts for approximately 40% of FALS cases and 5–10% of SALS. Other frequent ALS causative genes are *FUS* (5% FALS and < 1% SALS), and *TARDBP* (3% FALS and 2% SALS) [7,11,12,13,14,15]. In a percentage of ALS patients, more than one potentially pathogenic variant can be identified, suggesting an oligogenic basis of the disease with a dose-dependent gene-burden effect [16]. Recent studies proposed a multistep pathogenic model with subsequent intervening etiological events including genetic and environmental factors [17]. This model also implies that the number of steps necessary to start the neurodegenerative process in genetically mediated ALS may be reduced compared to cases without mutation, and the number of steps being variable according to the mutated gene [17,18]. Several ALS genes show pleiotropy and are associated with different clinical entities ranging from non-neurological diseases, such as glaucoma and bone diseases to ALS mimicking phenotypes such as motor neuropathies, spinal muscular atrophy, and distal myopathies [19]. Thus, it could be important to clarify whether other motor neuron disease genes may influence the pathology of genetic ALS [20]. In the view of the abundant and various genetic involvements in ALS, it is remarkable that a considerable percentage of individuals with positive family history remains without the identification of a genetic defect, suggesting a wider heterogeneity in the spectrum of the disease.

In this study we propose an NGS genetic approach to search for rare ALS gene variants in patients in which genetic defects in SOD1, FUS, TARDBP, and C9orf72 have been excluded. The aim is to reveal causative genetic factors related to ALS in a representative cohort of Italian patients, to contribute in the characterization of overlapping clinical phenotypes and improve accuracy in diagnosis and genetic counseling for this rare group of diseases.

## 2. Methods

### 2.1. Patients 

Patients participating in this study were 213 unrelated ALS Italian subjects (126 men, 59%). This comprised a retrospective consecutive series of patients in which *SOD1*, *FUS*, and *TARDBP* gene mutations and the hexanucelotide repeat expansions in *C9orf72* were previously excluded. Mean age at genetic screening was of 59 ± 13 years. Thirty-four index subjects (16%) reported a positive family history for motor neuron disease. The Ethics Standard Committee of our institution approved the study, and written informed consent was obtained from all participants.

### 2.2. Genetic Screening 

Genomic DNA was extracted from venous peripheral blood lymphocytes by standard procedures. Preliminary mutational screening for *SOD1*, *FUS*, *TARDBP*, and *C9orf72* genes variants was performed as previously described [10,13,14]. Next-generation sequencing was performed by either one of the following custom gene panels: a) amplicon-based customized NGS panel (Illumina TrueSeq Custom Amplicon, TSCA) covering 48 ALS/FTD genes (143 index cases); b) probe-based customized panel (Illumina Nextera Rapid Capture Custom kit, Illumina) covering 78 genes, including ALS/FTD causative and susceptibility genes, hereditary motor neuronopathy (HMN), and hereditary spastic paraplegia (HSP) genes (71 index cases). For the purposes of the study, the same set of 46 genes common for the two NGS panels were analyzed: *ALS2*, *ANG*, *APEX1*, *ASAH1*, *ATXN2*, *BSCL2*, *CHCHD10*, *CHMP2B*, *CRYM*, *CYP27A1*, *DAO*, *DCTN1*, *DPP6*, *ELP3*, *EPHA4*, *ERBB4*, *FIG4*, *FUS*, *GARS1*, *GRN*, *HNRNPA1*, *HNRNPA2B1*, *HNRNPA3*, *IGHMBP2*, *MAPT*, *MATR3*, *NEFH*, *NEK1*, *OPTN*, *PFN1*, *PNPLA6*, *REEP1*, *SETX*, *SIGMAR1*, *SOD1*, *SPAST*, *SPG11*, *SQSTM1*, *TAF15*, *TARDBP*, *TREM2*, *TRPV4*, *TUBA4A*, *UBQLN2*, *VAPB*, *VCP* (Table 1). The prescreened *SOD1*, *FUS*, *TARDBP* genes were also included in the gene sequencing panel, as internal control. Reference sequences for transcript (NM) and protein (NP) are reported in Supplementary Table S1.

### 2.3. Targeted Next-Generation Sequencing and Bioinformatic Analysis

Sequencing of enriched libraries was performed using an Illumina MiSeq sequencing device with paired end approach. Data analysis was performed using: a) MiSeq Reporter software (Illumina), for quality control, alignment against hg19 reference genome and variant calling; b) VariantStudio software (Illumina), public variation databases (Single Nucleotide Polymorphism database 137 (https://www.ncbi.nlm.nih.gov/projects/SNP), NHLBI Exome Sequencing Project 6500 (http://evs.gs.washington.edu/EVS), 1000 Genomes project (http://www.internationalgenome.org/1000-genomes-browsers/), Exome Aggregation Consortium (Exome Aggregation Consortium) and Human Gene Mutation Pro Database (http://www.hgmd.cf.ac.uk/ac/index.php) and an in-house database, for variants annotation; c) CLC Genomics Workbench software (CLCbio, Qiagen), for quality control and coverage analysis. 

Variants were filtered with the following criteria: (i) variants in the coding region or in the flanking 20 bp that were (ii) absent or rare with minor allele frequency (MAF) < 1% in the population databases (dbSNP137, NHLBI E SP6500, 1000 Genomes project, and ExAC). 

Taking into account the very low prevalence of ALS disease, the genetic variants with frequency > 1% in dbSNP or in Exome Variant Server or ExAC were classified as Class-1, and were escluded from subsequent analyses. In silico prediction of synonymous or intronic variants effect on splice site was performed using at least three splice prediction tools: NNSplice predictor; http://www.fruitfly.org/seq_tools/splice.html; http://wangcomputing.com/assp/. Best candidate variants were verified using Sanger methods. In subject heterozygous for a single likely pathogenic gene variant in recessive genes, we screened exon and intron-exon boundary sequences and gene copy number to search for a possible second mutation.

The genetic variants passing the filtering process were classified as “pathogenic” (Class-5), “likely pathogenic”(Class-4), “variants of unknown significance” (VUS, Class-3), and “unlikely pathogenic variants” (Class-2), according to criteria derived from a modified version of the classification proposed by Antoniadi et al. [21] (Supplementary Table S2).

## 3. Results

We identified a total of 160 variants in 117 out of 213 index cases (55%).

Ninenteen variants (17%) were classified as pathogenic (*n* = 11) or likely pathogenic (*n* = 8). Ten of these latter variants (Class 4–5) were in autosomal dominant ALS genes: *OPTN* (2), *VCP* (2); *SQSTM1* (2) *SETX* (1), *GARS1* (1), *FIG4* (2). The remaining nine were heterozygous variants identified in autosomal recessive genes: *ALS2* (1), *SIGMAR1* (1), *ASAH1* (1), *IGHMBP2* (5), *PNPLA6* (1). These patients screened negative for a second mutation in exon and intron-exon boundary sequences or gene copy number variants.

We also identified forty-nine VUS (44%, Class-3), and 44 unlikely pathogenic variants (39%, Class-2). No gene variants were detected in *APEX1*, *BSCL2*, *CHCHD10*, *HNRNPA1*, *HNRNPA2B1*, *PFN1*, *REEP1*, *TAF15*, and *TUBA4* genes, and, in the prescreened *FUS*, *SOD1*, *TARDBP* genes (Table 2).

Forty-eight variants were found in ALS susceptibility genes (Table 3). Six variants were recurrent: the c.1472A>G in *ATXN2* (2 patients), the synonimous *ATXN2* variant c.3000A>G (7 patients), the c.47A>C in *CRYM* gene (2 patients), the c.3529 + 5G>A in *DCTN1* (3 patients)*,* the c.196-7T>G in *HNRNPA3* (3 patients), and the c.1054C>A *NEFH* gene variant (4 patients) (Supplementary Table S3).

After classification of all gene variants, the patients were stratified according to the carried variant with the highest pathogeneicity class in ALS/FTD/HMN-HSP genes. 

Nine patients carried pathogenetic or likely patogenetic variants in ALS/HMN genes (4.2%), 18 subjects carried heterozygous Class-3 variant in autosomal dominant or X-linked ALS genes (8.4%), and 12 patients (5.6%) had heterozygous variants in FTD/HMN-HSP genes. Eleven patients showed the coexistence of multiple variants of Class ≤ 3. Only one Class-3 variant *(GARS1* c.303G>A; p.Arg101His) was recurrent in two patients. 

**Figure 1:**
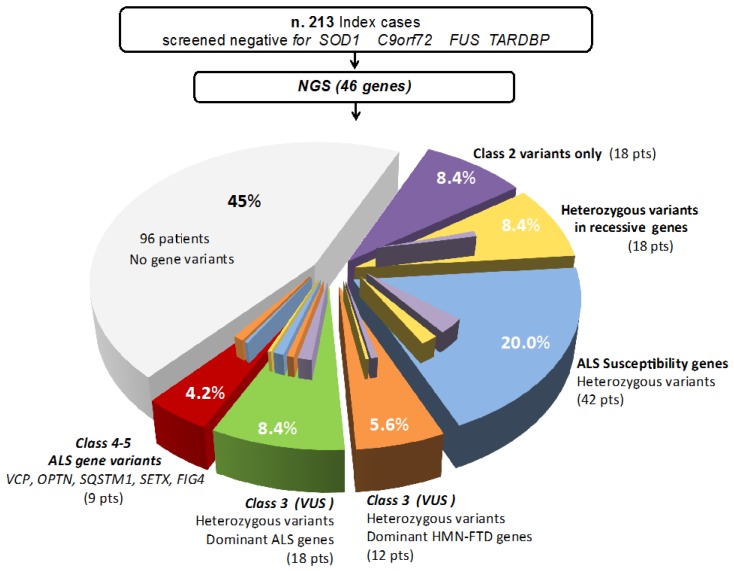
Gene variants and their estimated pathogeneicity class in a cohort of 213 Italian ALS patients tested negative for the most frequent ALS causative genes. Values represent the relative contribution of mutations in ALS genes, other motor neuron disease genes, and gene associated with frontotemporal dementia (FTD).

Fourty-two patients (20%) carried only variants in ALS susceptibility genes (Supplementary Table S3); 18 subjects (8.4%) carried heterozygous variant in autosomal recessive ALS genes (Supplementary Table S4), and others 18 patients (8.4%) had only Class-2 variants (Supplementary Table S5). None of the index patient had more than one pathogenic or likely pathogenic variants in autosomal dominant ALS genes. No recurrent Class 4-5 variants were identified in our series (Figure 1).

### Clinical Phenotypes of Patients with Class-3 to Class-5 Gene Variants 

For 9 patients carrying Class-4 and Class-5 gene variants, the molecular findings supported a probable diagnosis of genetic ALS: two were subjects carrying different heterozygous Class-5 missense variants in *OPTN*, two subjects had different pathogenic Class-5 variants in *VCP* gene, one patient was compound heterozygous for a Class-4 and a Class-5 *FIG4* variant (P100), two subjects carried a Class-4 missense variant and a splicing variant in *SQSTM1* (P002, and P103), and one subject carried a Class-4 heterozygous *GARS1* gene variant (c.1955G>A; p.Gly652Glu, P073) (Table 4). Thirty patients carried variants of unknown significance (Class-3) in ALS/HMN/FTD genes. For these patients, a definite genetic cause for the disease could not be ascertained (Table 5).

#### OPTN Gene

A 60 year old patient (P087) was positive for the previously described p.Gln314Leu missense heterozygous variant (Class-5) in the *OPTN* gene [22]. The patient presented, at age 59, progressive bulbar deficit with functional impairment of swallowing and speech. Neuropsychological evaluation showed mild depression and emotional liability. EMG showed active denervation in the bulbar district and hand muscles. The *OPTN* gene variant p.Gln314Leu has been described so far in two patients with motor neuron disease [22,23], and in 1/400 control subjects aged >56 years. In our “in house” database, we only observed this genetic variant in one unrelated ALS subject and in none of the 280 controls [22]. Another patient (P015) carried the previously described p.Ala481Val *OPTN* Class-5 missense variant. The patient was a 61-year old man presenting muscle cramps, fasciculation, increased creatine kinase (CK), progressive muscle weakness, and diffuse hyperreflexia. His mother had the diagnosis of Parkinson disease. This variant was previoulsy described in an heterozygous patient with ALS [24] and in an FTD patient who was a compound heterozygote for the p.Gln235* nonsense and the p.Ala481Val mutation [25]. A third patient carried a novel Class-3 *OPTN* variant, p.Leu304Phe, predicted to be deleterious at SIFT (Sorting Intolerant From Tolerant)and PolyPhen-2 prediction programs. The patient (P075) presented spasticity and muscle weakness and wasting at the age of 72, and was dignosed as having ALS at age 75. EMG showed diffuse signs of active denervation. The mother was referred to be affected by Alzheimer disease. 

#### VCP Gene

Two patients carried previously described Class-5 heterozygous *VCP* variants: the p.Arg93Cys [26,27] and the p.Arg155Cys [28,29]. The patient carrying the p.Arg93Cys *VCP* variant (P072), presented gait difficulties at age of 47. The disease had a progressive course and at age 60 he was not able to walk independelty, had severe muscle wasting in four limbs. No bubar signs or cognitive decline were reported, but the mother and two maternal uncles were diagnosed with Alzheimer disease. A brother had the diagnosis of ALS and Paget disease.The patient carrying the p.Arg155Cys *VCP* gene variant (P008) had an early onset of the disease at 42 years. He presented severe and diffuse muscle weakness, muscle wasting, and fasciculations. CK level was 255 U/L (n.v. 24–195). EMG showed chronic neurogenic changes and active denervation. The mother and a maternal aunt were reported to have mild muscle weakness at lower limbs, but no specific diagnosis was made. The patient had no bone alterations or cognitive impairement. Neuropsychological tests reveal only mild deficit of episodic memory. Brain MRI was normal and the muscle biopsy showed a few fibers with rimmed vacuoles and cores. Two other patients carried Class-3 *VCP* variants: a 69 year old woman (P112), heterozygous for the missense p.Lys60Arg *VCP* variant, presented slowly progressive bulbar and limb muscle weakness and spasticity associated with cognitive decline. The second patient (P025), a 76 year old man heterozygous for the c.1696-3C>T *VCP* splicing variant, presented diffuse muscle weakness and wasting, cognitive and behavioral difficulties. He died one year later from disease complications.

#### SQSTM1 Gene

Two subjects aged 43 and 74, (P002, and P103) carried likely pathogenic variants (Class-4) in *SQSTM1* gene. Both patients, aged 43 and 74, were sporadic cases and presented with clinical features consistent with the diagnosis of possible ALS (Table 4). A third patient carried a Class-3 *SQSTM1* gene variant. Her presenting phenotype was characterized by diffuse and severe muscle weakness, but subsequent clinical evaluations excluded ALS disease and suggested a possible diagnosis of acquired myopathy.

#### FIG4 Gene

In a 27-year-old female patient (P100), genetic screening revealed a compound heterozygosity for two *FIG4* missense variants: c.122T>C (p.Ile41Thr) and c.1667C>T (p.Thr556Ile). She had a clinical diagnosis of juvenile ALS with predominant upper motor neuron involvement. Clinical and molecular data of this patient was already reported by Bertolin et al. [30]. The c.122T>C pathogenic variant (Class-5) was previously reported in compound heterozygosis in a patient affected by Charcot–Marie–Tooth disease type 4J [31,32]. The c.1667C>T is a novel variant that causes the substitution of a highly conserved amino acid and was classified as Class-4. In silico studies predicted a damaging effect on protein function. *FIG4* variants of Class-3 were detected in two patients. Both patients were sporadic and had different phenotypes: one subject, aged 64, presented progressive muscular wasting and peripheral neuropathy (P105), and the second case was a 39 year old woman with spastic quadriplegia since the age of 29 (P106).

#### SETX Gene

In patient P022, we identified a *SETX* Class-4 variant (c.2750T>C; p.Met917Thr) that was previously reported in an ALS patient [5]. Our patient was a 71-year-old woman presenting a bulbar onset of the disease and a severe and progressive muscular weakness and wasting. Six additional patients carried Class-3 *SETX* variants (Table 5). All patients were sporadic and 5 out of 6 had a classical ALS phenotype with ages at onset ranging from 43 to 79 years. One patient (P071) had a juvenile onset of slowly progressive lower motor neuron involvement.

#### GARS1 Gene 

A rare missense likely pathogenic variant was identified in the *GARS1* gene (c.1955G>A; p.Gly652Glu) predicted to be deleterious (PolyPhen-2: probably_damaging; SIFT: deleterious). Patient P073 was first evaluated at 58 years because of right foot drop without pain or sensory abnormalities. EMG showed active denervation in right tibialis anterior and extensor digitorum, with normal distal conduction velocities. Lumbar MRI showed foraminal stenosis at L3-L4, L4-L5, and L5-S1. At last evaluation, four years after symptom onset, electrophysiological studies showed severe motor axonopathy at lower limbs with active and chronic denervation signs. Motor evoked potentials disclosed reduction of the amplitude with normal latencies at four limbs, while sensory evoked potentials were normal. Based on neurophysiological data and signs of upper motor neuron involvement, a diagnosis of ALS pseudopolyneuritic type was made. Four additional patients carried *GARS1* heterozygous missense variants classified as VUS. These subjects showed involvement of both upper and lower motor neurons, and one patient had bulbar onset ALS associated with FTD.

## 4. Discussion and Conclusion

Our knowledge about the genetic of ALS has significantly improved over the last years and the presence of causative genetic variants has been extensively investigated in different populations. The most frequently recognized disease causing mutations are found in *C9orf72*, *SOD1*, *TARDBP*, and *FUS* genes. In the patients referred to our center, including more than 400 ALS subjects, the repeat expansion in the *C9orf72* gene is detected in 7.3% of cases, *SOD1* gene variants in 4%, *TARDBP* in 1%, and *FUS* gene variants in 0.2% of cases. Similar percentages of causative variants in these major ALS genes were reported in other series of ALS patients [33]. In the present study, we selected 213 consecutive ALS patients negative for mutations in *SOD1*, *FUS*, *TARDBP*, and *C9orf72*, and we performed an extensive targeted NGS screening to diagnose rare forms of genetic ALS. The ALS/FTD associated genes that have been examined represent an almost complete analysis of the current list, excluding TBK1. A major difficulty in NGS analyses was the interpretation of the pathogenicity of the novel variants. Our variant classification was based on a modified version of the criteria proposed by Antoniadi et al. [21] for the diagnosis of inherited peripheral neuropathies with more restrictive criteria for the frequency of the reported variants. 

Our classification consideres most of the criteria recommeded in the guidelines for the interpretation of sequence variants privided by the American College of Medical Genetics and Genomics (ACMG) [34], including population databases, in silico prediction tools, in-house variant database, and the appropriate scientific literature. However, due to predominance of late onset sporadic ALS cases in our cohort, we could not reliably count on the criteria based of segregation analysis and functional studies.

Our study allowed the identification of pathogenic or likely pathogenic variants in 4.2% of patients. This result is consistent with that reported in other high-throughput sequencing studies in which the contribution of the major ALS genes was excluded. 

In fact, despite the large number of ALS screened genes and the vast number of identified variants, the diagnosis of rare genetic forms of ALS usually ranges from 4% to 5% of cases [35,36,37,38]. In recent studies on ALS patients from different populations, rare gene variants were reported in *SETX* (0.3–2%), *ANG* (0–1%), *OPT* (0.2–1%), *ALS2* (0.3–1.5%), *VCP* (0.2–0.3%), *UBQLN2* (0–0.7%), *CHCHD10* (0.7%), *VAPB* (0–0.3%), and *NEFH* (0–0.3%) [35,36,37,38].

In our survey, the genes with the highest percentage of pathogenic variants were *OPTN*, *VCP*, and *SQSTM*1.

Mutations in *OPTN* were firstly reported to be causative of a primary open-angle glaucoma (MIM:137760), and subsequently Maruyama et al. [39] described different types of mutations (both homozygous and heterozygous) in ALS. We identified two *OPTN* Class-5 variants (1%), the p.Gln314Leu [22,23] and the p.Ala481Val [24,25]. The first *OPTN* gene variant was previously detected in two unrelated ALS patients, and in 1 out of 400 control subjects. The occurrence of the variant in a patient from our series seems to confirm its pathogenic role, although variable penetrance could also been considered [23]. The second p.Ala481Val *OPTN* variant was previously described in an heterozygous ALS patient from a Canadian cohort [24], and in a 64-year-old patient with FTD from a different study [25]. This latter subject was a compound heterozygote for two *OPTN* variants including the missense p.Ala481Val and the p.Gln235* nonsense mutation [25]. Our 61 year-old patient was a sporadic case with a clinical phenotype resembling that of the patient reported by Belzil et al., being primarely characterized by spinal ALS without evidence of cognitive impairment [24].

Two patients (1%) carried previously described Class-5 heterozygous *VCP* variants: the p.Arg93Cys [26,27] and the p.Arg155Cys [28,29]. Mutations in the valosin-containing protein, an ATPase involved in protein degradation and autophagy, have previously been identified in families with inclusion body myopathy, Paget’s disease, and frontotemporal dementia (IBMPFD, MIM:167320). Johnson et al. [40] described for the first time *VCP* mutations in ALS cases, including patients belonging to a four-generation Italian family. In a cohort of 231 individuals carrying *VCP* mutations, Al-Obeidi et al. reported myopathy in 90% of patients, Paget’s disease of bone in 42%, frontotemporal dementia in 30%, and ALS phenotype in approximately 9% of patients [41]. The phenotype in our two cases was consistent with previously described patients with unequivocal upper and lower motor neuron involvement, at the ages of 47 and 42 years, without FTD or Paget’s disease. The *VCP* p.Arg93Cys variant, identified in patient P072, was previously reported in a 70-year-old patient with a diagnosis of distal myopathy and rimmed vacuoles [27], and in several subjects from a single family in which three subjects presented cognitive impairement and myopathy, and three others had Paget’s disease [26]. In our case, the mother and two maternal uncles were diagnosed with Alzheimer disease, and a brother had the diagnosis of ALS and Paget disease. The second Class-5 *VCP* variant (p.Arg155Cys) was also reported in several families with IBMPFD [28,29]. The clinical phenotype of our patient (P008) was characterized by progressive motor neuron impairement and, interestingly, rimmed vacules at muscle biopsy morphological evaluation.

The third gene responsible for ALS disease in our study was *SQSTM1* (1%). Also, this gene has been associated with a wide spectrum of phenotypes. In 2002, a recurrent non-conservative change in *SQSTM1* (p.Pro392Leu) was reported in a high proportion of French Canadian patients affected by Paget disease [42]. Subsequently heterozygous mutations in *SQSTM1* were reported in patients presenting ALS, in patients with ALS, and FTD or Paget disease, and in a patient with isolated distal rimmed-vacuole myopathy [43,44,45,46,47]. In our *SQSTM1* mutated patients, the clinical phenotype was characterized by typical ALS disease, with no signs of bone abnormalities or FTD.

Finally, pathogenic variants were identified in the autosomal dominant genes *SETX* (0.4%), *FIG4* (0.4%), and *GARS1* (0.4%). Heterozygous missense variants in *SETX* were suggested to cause a rare autosomal dominant form of juvenile ALS (MIM:602433) with onset <25 years and a slow rate of disease progression [48]. In our cohort, we identified seven *SETX* gene variants: six were classified as VUS, and the p.Met917Thr variant was classified as likely pathogenic (Class-4) because already reported in an ALS patient [5]. The authentic contribution of this variant to ALS disease, as well as for other *SETX* heterozygous variants reported in ALS, remains to be further investigated and proven. The p.Met917Thr *SETX* variant is reported in population databases, and in our case, was associated with a late onset and rapidly progressive ALS phenotype. In several NGS studies, there is an abundance of *SETX* variants, mostly identified in sporadic with the typical late-onset ALS phenotype [5,35,49]. Due to the relatively high detection rate of heterozygous *SETX* variants both in patients and controls, it is still controversial if this gene may contribute to a substantial proportion of genetic ALS or if it should be rather considered as a susceptibility genetic factor. A clear pathogenic role is demonstrated for biallelic mutations in *SETX* for a recessive form of cerebellar ataxia with oculomotor apraxia type 2 (AOA2, MIM:606002). In AOA2 families, an increase incidence of ALS disease in *SETX* heterozygous family members has not been reported. 

*GARS1* and *FIG4* genes are both associated with either ALS or hereditary neuropathy [31,50,51,52]. Clinical and neurophysiological data of our patients confirmed the involvement of both upper and lower motor neurons, supporting the association with the ALS phenotype. 

We also reported the identification of 49 novel or rare variants in 19 different genes, whose pathogenetic significance could not be presently determined. These VUS involved 30 patients for which genetic counseling remains challenging and genetic informations might need periodic reevaluation. The same difficulties can be shared for the 48 variant identified in ALS susceptibility genes. For *ATXN2*, we describe missense variants, but an increased risk of developing ALS disease has been associated, so far, only with alleles carrying long sequences of repeated CAG triplets [53]. Thus, the possible role in ALS susceptibility for rare missense variants within other coding regions is not yet defined. With the advent of NGS technology, an increasingly number of rare genetic variants are being identified in known ALS genes and the genetic architecture of ALS is emerging in all its complexity. Even though the use of a targeted panel is cost effective to screen a large list of genes, the classification of all identified variants and segregation studies are critical procedures requiring time and resources. For timely diagnoses and genetic counseling, it could be useful to consider the reported frequencies of causative genes to prioritize the analyses of rare pathogenic variants.

The results of our study highlight the relative contribution of *OPTN*, *VCP*, and *SQSTM1* variants in rare forms of genetic ALS, suggesting the screening of these genes during routine diagnostic investigations in both sporadic and familial ALS cases.

## Figures and Tables

**Table 1:** Genes analyzed in this study.

ALS	Gene	Protein	Disease[OMIM]	Inherit.
**ALS1**	*SOD1*	Superoxide Dismutase [Cu-Zn]	Amyotrophic lateral sclerosis 1 [MIM:105400]	AD
			Amyotrophic lateral sclerosis 1 [MIM:105400] D90A; D96N	AR
**ALS2**	*ALS2*	Alsin	Amyotrophic lateral sclerosis 2 [MIM:205100]	AR
			Infantile-onset ascending spastic paralysis (IAHSP) [MIM:607225]	AR
			Juvenile primary lateral sclerosis (JPLS) [MIM:606353]	AR
**ALS4**	*SETX*	Probable helicase senataxin	Amyotrophic lateral sclerosis 4, juvenile ALS [MIM:602433]	AD
			Spinocerebellar ataxia, 1 (SCAR1) [MIM:606002]	AR
**ALS5**	*SPG11*	Spatacsin	Amyotrophic lateral sclerosis 5, juvenile [MIM:602099]	AR
			Charcot–Marie–Tooth disease, axonal type 2X(CMT2X) [MIM:616668]	AR
			Spastic paraplegia 11, (SPG11) [MIM:604360]	AR
**ALS6**	*FUS*	RNA-binding protein FUS	Amyotrophic lateral sclerosis 6, +/- FTD [MIM:608030]	AD
			Tremor, hereditary essential 4 (ETM4) [MIM:614782]	AD
**ALS8**	*VAPB*	Vesicle-associated membrane protein-B/C	Amyotrophic lateral sclerosis 8 [MIM:608627]	AD
			Spinal muscular atrophy, late-onset, (SMAFK) [MIM:182980]	AD
**ALS9**	*ANG*	Angiogenin	Amyotrophic lateral sclerosis 9; [MIM:611895]	AD
**ALS10**	*TARDBP*	TAR DNA-binding protein 43	Amyotrophic lateral sclerosis 10, +/- FTD [MIM:612069]	AD
**ALS11**	*FIG4*	Polyphospho inositide phosphatase	Amyotrophic lateral sclerosis 11 [MIM:612577]	AD
			Charcot–Marie–Tooth disease 4J (CMT4J) [MIM:611228]	AR
			Polymicrogyria, bilateral temporooccipital (BTOP) [MIM:612691]	AR
**ALS12**	*OPTN*	Optineurin	Amyotrophic lateral sclerosis 12 [MIM:613435]	AR/AD
			Glaucoma 1, open angle, E (GLC1E) [MIM:137760]	AD
**ALS14**	*VCP*	Transitional endoplasmic reticulum ATPase	Amyotrophic lateral sclerosis 14, +/- FTD [MIM:613954]	AD
			Charcot–Marie–Tooth disease, type 2Y (CMT2Y) [MIM:616687]	AD
			Inclusion body myopathy with Paget disease +/- FTD [MIM:167320]	AD
**ALS15**	*UBQLN2*	Ubiquilin-2	Amyotrophic lateral sclerosis 15, +/- FTD [MIM:300857]	XLD
**ALS16**	*SIGMAR1*	Sigma non-opioid intracellular receptor 1	Amyotrophic lateral sclerosis 16, juvenile [MIM:614373]	AR
			Distal spinal muscular atrophy, 2 (DSMA2) [MIM:605726]	AR
**ALS17**	*CHMP2B*	Charged multivesicular body protein 2b	Amyotrophic lateral sclerosis 17 [MIM:614696]	AD
			FTD chromosome 3-linked (FTD3) [MIM:600795]	AD
**ALS18**	*PFN1*	Profilin-1	Amyotrophic lateral sclerosis 18 [MIM:614808]	AD
**ALS19**	*ERBB4*	Receptor tyrosine-protein kinase erbB-4	Amyotrophic lateral sclerosis 19 [MIM:615515]	AD
**ALS20**	*HNRNPA1*	Heterogeneous nuclear ribonucleoprotein A1	Amyotrophic lateral sclerosis 20 [MIM:615426]	AD
			Inclusion body myopathy with Paget disease +/- FTD3 [MIM:615424]	AD
**ALS21**	*MATR3*	Matrin-3	Amyotrophic lateral sclerosis 21 [MIM:606070]	AD
**ALS22**	*TUBA4A*	Tubulin alpha-4A chain	Amyotrophic lateral sclerosis 22, +/- FTD [MIM:616208]	AD
**FTDALS2**	*CHCHD10*	Coiled-coil-helix-coiled-coil-helix domain-containing protein 10, mitochondrial	FTD +/- amyotrophic lateral sclerosis 2 [MIM:615911]	AD
			Isolated Mitochondrial Myopathy (IMMD) [MIM:616209]	AD
			Spinal muscular atrophy, Jokela type (SMAJ) [MIM:615048]	AD
**FTDALS3**	*SQSTM1*	Sequestosome-1	FTD +/- amyotrophic lateral sclerosis 3 [MIM:616437]	AD
			Ataxia, dystonia, gaze palsy[MIM:617145]	AR
			Myopathy distal, with rimmed vacuoles [MIM:617158]	AD
			Paget disease of bone 3 (PDB3) [MIM:167250]	AD
**ALS** suscep	*ATXN2*	Ataxin-2	{Amyotrophic lateral sclerosis, susceptibility to, 13} [MIM:183090]	AD
			Spinocerebellar ataxia 2 (SCA2) [MIM:183090]	AD
**ALS** suscep	*APEX1*	apurinic/apyrimidinic endodeoxyribonuclease1		
**ALS** suscep	*CRYM*	Ketimine reductase mu-crystallin	Deafness, autosomal dominant 40 (DFNA40) [MIM:616357]	AD
**ALS** suscep	*CYP27A1*	Sterol 26-hydroxylase,	Cerebrotendinous xanthomatosis (CTX) [MIM:213700]	AR
**ALS** suscep	*DAO*	D-amino-acid oxidase		
**ALS** suscep	*DCTN1*	Dynactin subunit 1	{Amyotrophic lateral sclerosis, susceptibility to} [MIM:105400]	
			Neuronopathy, distal hereditary motor, 7B (HMN7B) [MIM:607641]	AD
			Perry syndrome (PERRYS) [MIM:168605]	AD
**ALS** suscep	*DPP6*	Dipeptidyl-aminope-peptidase-like protein 6	Mental retardation, autosomal dominant 33 (MRD33) [MIM:616311]	AD
**ALS** suscep	*ELP3*	Elongator Acetyltransferase Complex Subunit 3		
**ALS** suscep	*EPHA4*	Ephrin type-A receptor 4		
**ALS** suscep	*HNRNPA2B1*	Heterogeneous nuclear ribonucleoproteinsA2/B1	Inclusion body myopathy and Paget disease +/- FTD [MIM:615422]	
**ALS** suscep	HNRNPA3	Heterogeneous nuclear ribonucleo-protein A3		
**ALS** suscep	*NEFH*	Neurofilament heavy polypeptide	{Amyotrophic lateral sclerosis, susceptibility to} [MIM:105400]	
			Charcot–Marie–Tooth, axonal, type 2CC (CMT2CC) [MIM:616924]	AD
**ALS** suscep	*NEK1*	Serine/threonine-protein kinase Nek1	Short-rib thoracic dysplasia 6 +/- polydactyly(SRTD6) [MIM:263520]	AR
**ALS** suscep	*TAF15*	TATA-binding protein-associated factor 2N	Chondrosarcoma, extraskeletal myxoid [MIM:612237]	
**ALS** suscep	*TREM2*	Triggering receptor expressed myeloid cells2	Polycystic lipomembranous osteodysplasia with sclerosing leukoencephalopathy (PLOSL) [MIM:221770]	AR
**FTD**	*GRN*	Granulins	FTD with ubiquitin-positive inclusions ([MIM:607485]	AD
			Aphasia, primary progressive [MIM:607485]	AD
			Ceroid lipofuscinosis, neuronal, 11 (CLN11) [MIM:614706]	AR
**FTD**	*MAPT*	Microtubule-associated protein tau	Frontotemporal dementia (FTD) [MIM:600274]	AD
			Pick disease of the brain (PIDB) [MIM:172700]	AD
			Progressive supranuclear palsy 1 (PSNP1) [MIM:601104]	AD
			Parkinson-dementia syndrome (PARDE) [MIM:260540]	AR
**SMA**	*ASAH1*	Acid ceramidase	Farber lipogranulomatosis (FRBRL) [MIM:228000]	AR
			Spinal muscular atrophy, progressive myoclonic epilepsy[MIM:159950]	AR
**HMN/HSP**	*BSCL2*	Seipin	Lipodystrophy congenital generalized type 2 (CGL2) [MIM:269700]	AR
			Encephalopathy progressive, +/- lipodystrophy (PELD) [MIM:615924]	AR
			Neuronopathy, distal hereditary motor, 5A (HMN5A) [MIM:600794]	AD
			Spastic paraplegia 17, (SPG17) [MIM:270685]	AD
**HMN**	*GARS1*	Glycyl-tRNA synthetase 1	Charcot-Marie-Tooth disease 2D (CMT2D) [MIM:601472]	AD
			Neuronopathy, distal hereditary motor, 5A (HMN5A) [MIM:600794]	AD
**HMN**	*IGHMBP2*	Immunoglobulin mu-binding protein 2	Charcot–Marie–Tooth disease 2S (CMT2S) [MIM:616155]	AR
			Neuronopathy, distal hereditary motor, 6 (HMN6) [MIM:604320]	AR
**HMN/HSP**	*PNPLA6*	Neuropathy target esterase	Boucher–Neuhauser syndrome (BNHS) [MIM:215470]	AR
			Laurence-Moon [MIM:245800];Oliver-McFarlane [MIM:275400]	AR
			Spastic paraplegia 39 (SPG39) [MIM:612020]	AR
			Neuronopathy, distal hereditary motor, 5B (HMN5B) [MIM:614751]	AR
**HSP**	*REEP1*	Receptor expression-enhancing protein1	Spastic paraplegia 31 (SPG31) [MIM:610250]	AD
**HSP**	*SPAST*	Spastin	Spastic paraplegia 4 (SPG4) [MIM:182601]	AD
**HMN**	*TRPV4*	Transient receptor potential cation channel subfamily V4	Digital arthropathy-brachydactyly, familial (FDAB) [MIM:606835]	AD
			Neuronopathy, distal hereditary motor, 8 (HMN8) [MIM:600175]	AD
			Scapuloperoneal spinal muscular atrophy (SPSMA) [MIM:181405]	AD

ALS suscep: amyotrophic lateral sclerosis susceptibility genes; SMA: spinal muscular atrophy; HMN: hereditary motor neuronopathy; HSP: hereditary spastic paraplegia; FTD frontotemporal dementia; MIM, Mendelian Inheritance in Man; IBMPFD inclusion body myopathy and Paget disease +/- FTD; AD, autosomal dominant; AR, autosomal recessive; XLD, X-linked dominant. +/- with or without.

**Table 2:** Summary of identified gene variants and classes of pathogenicity.

	Gene	N. of Variants	Class 5	Class 4	Class 3	Class 2	Recurrent Variants
**ALS (AD)**	*ANG*	4			1	3 *	* 1 recurrent in 3 pts
	*CHCHD10*	0					
	*CHMP2B*	1			1		
	*ERBB4*	4			2	2 *	* 1 recurrent in 2 pts
	*FIG4*	4	1	1	2		
	*HNRNPA1*	0					
	*MATR3*	1			1		
	*OPTN*	3	2		1		
	*PFN1*	0					
	*SETX*	21		1	6	14 *	* 1 recurrent in 7 pts * 1 recurrent in 5 pts
	*SQSTM1*	6		2	1	3 *	* 1 recurrent in 3 pts
	*TUBA4A*	0					
	*VAPB*	3				3 *	* 1 recurrent in 3 pts
	*VCP*	4	2		2		
**ALS (XLD)**	*UBQLN2*	1			1		
**ALS (AR)**	*ALS2*	4		1	3		
	*SIGMAR1*	1		1			
	*SPG11*	7			3	4	
**HMN/HSP** **(AD)**	*BSCL2*	0					
	*GARS1*	9		1	5 ^§^	3 *	^§^ 1 recurrent in 3 pts * 1 recurrent in 3 pts
	*REEP1*	0					
	*TRPV4*	9			3	6 *	* 1 recurrent in 3 pts
	*SPAST*	1				1	
**SMA/HMN/HSP** **(AR)**	*ASAH1*	3	1		2		
	*IGHMBP2*	10	4	1	4	1	
	*PNPLA6*	6	1		5		
**FTD (AD)**	*GRN*	8			4	4*	* 1 recurrent in 4 pts
	*MAPT*	2			2		
**Total**		**112**	**11**	**8**	**49**	**44**	10 recurrent variants (9 Class-2, 1 Class-3)

Specific DNA and protein variants of Classes 3-4-5 are listed in Table 4; Table 5. Class-2 variants are listed in Supplementary Table S5. ^§^: recurrent variants of class 3; *: recurrent variants of class 2.

**Table 3:** Total gene variants identified in amyotrophic lateral sclerosis (ALS) susceptibility genes.

	Gene	Number of Variants	
**ALS susceptibility genes**	*APEX1*		
	*ATXN2*	14 *	* 1 recurrent in 2 pts * 1 recurrent in 7 pts
	*CRYM*	2 *	* 1 recurrent in 2 pts
	*CYP27A1*	4	
	*DAO*	1	
	*DCTN1*	10 *	* 1 recurrent in 3 pts
	*DPP6*	1	
	*ELP3*	2	
	*EPHA4*	2	
	*HNRNPA2B1*		
	*HNRNPA3*	5 *	* 1 recurrent in 3 pts
	*NEFH*	4 *	* 1 recurrent in 4 pts
	*NEK1*	1	
	*TAF15*		
	*TREM2*	2	
	**Total**	**48**	**6 recurrent variants**

Specific DNA and protein variants in susceptibility genes are listed in Supplementary Table S3. *: recurrent variants.

**Table 4:** Pathogenic and likely pathogenic variants in ALS genes (Classes 4 and 5).

Gene	Class	Variant DNA	Variant Protein	MAF % *	dbSNP	Additional Variants	Clinical Notes	Patient	Ref.
***OPTN***	**5**	c.941A>T	p.Gln314Leu	0%-0.02%-0.01%	rs142812715	(**AR**) *ALS2*: c.331G>A (p.Val111Ile) het [rs61745503, MAF *: 0.04%-0.02%-0.03%] (Class3)	Bulbar-Spinal ALS laboratory supported	P087	[22,23]
***OPTN***	**5**	c.1442C>T	p.Ala481Val	0%-0.01%-0%	rs377219791		Spinal ALS-laboratory supported	P015	[24,25]
***VCP***	**5**	c.277C>T	p.Arg93Cys	0%-0%-0%			Spinal ALS, no FTD, no Paget	P072	[26,27]
***VCP***	**5**	c.463C>T	p.Arg155Cys	0%-0%-0%	rs121909330	(**AR**) *IGHMBP2*: c.2911_2912delAG (p.Arg971GlufsTer4) het [rs572973851; rs724159994, MAF *: 0%-0.82%-0.02%] (Class5)	Spinal ALS, no FTD, no Paget	P008	[28,29]
***SQSTM1***	**4**	c.695C>T	p.Pro232Leu	0%-0%-0%	rs757778292	(**Suscep.**) *DAO*: c.627G>A (p.Trp209Ter) het [rs766258671, MAF*: 0%-0%-0% ] (**Suscep.**) *DCTN1*: c.652G>A(p.Glu218Lys) het [MAF *: 0%-0%-0%]	Bulbar ALS, onset 43 years	P002	
***SQSTM1***	**4**	c.301 + 4delA	p.? HSF: Alteration of the WT donor site, most probably affecting splicing.	0%-0%-0%		(**AR**) *IGHMBP2*: c.2176G>A (p.Val726Met) het [rs143986510, MAF *: 0.02%-0.08%-0.04%] (Class3)	ALS	P103	
***FIG4***	**5** **4**	c.122T>C c.1667C>T	p.Ile41Thr p.Thr556Ile	0.08%-0.11%-0.1% 0%-0%-0%	rs121908287		Juvenile ALS with predominant upper motor neuron involvement	P100	[30]
***SETX***	**4**	c.2750T>C	p.Met917Thr	0%-0.01%-0.01%	rs376022544		ALS	P022	[5]
***GARS1***	**4**	c.1955G>A	p.Gly652Glu	0%-0%-0%	rs747080824		ALS Pseudo-polyneuritic type	P073	

* MAF (minor allele frequency) % in 1000Genomes-Go-ESP-ExAC. Het: heterozygous; HSF: Human Splicing Finder; (Suscep.): variants in ALS susceptibility genes; (AR): variants in autosomal recessive genes.

**Table 5:** Variants of unknown significance (VUS) in ALS/HMN/FTD genes (Class 3).

Gene	Class	Variant DNA	Variant Protein	MAF % *	dbSNP	Additional Variants	Clinical Notes	Patient
***OPTN***	**3**	c.910C>T	p.Leu304Phe	0%-0%-0%		(**Suscep.**) *ATXN2*: c.3000A > G (*p* =) het [rs140262591, MAF *: 0.2%-0.31%-0.3%]	Spinal ALS	P075
***VCP***	**3**	c.179A>G	p.Lys60Arg	0%-0%-0%		(**Suscep.**) *ATXN2*: c.3322C > T (p.Pro1108Ser) het [rs140242317, MAF *: 0.04%-0.11%-0.07% ]	Slowly progressive ALS with cognitive impairment	P112
***VCP***	**3**	c.1696-3C>T	p.?	0%-0.01%-0.01%	rs372638909		Rapidly progressive ALS with cognitive impairment	P025
***FIG4***	**3**	c.646G>A	p.Gly216Arg	0%-0%-0%	rs759566206	(**Class 2**) *SQSTM1*: c.712A > G (p.Lys238Glu) het [rs11548633, MAF *: 0.24%-0.26%-0.24%]	Progressive muscular weakness and sensory neuropathy	P105
***FIG4***	**3**	c.1243C>G	p.Pro415Ala	0%-0%-0%		(**Class 3**) *GARS1*: c.302G > A (p.Arg101His) het [rs200887429, MAF *: 0.02%-0.02%-0.04%]; (**Class 2**) *SETX*: c.4660T > G (p.Cys1554Gly) het [rs112089123, MAF *: 0.58%-0.31%-0.58%]	Spastic quadriplegia	P106
***SQSTM1***	**3**	c.833C>T	p.Thr278Ile	0%-0%-0%	rs200445838		Myopathy	P043
***UBQLN2***	**3**	c.809G>A hem	p.Arg270His	0%-0%-0%	rs767597171		Spinal ALS (Flail arm)	P017
***SETX***	**3**	c.-114-2A>G	p.?	0.08%-0%-0%	rs560095915		Bulbar ALS, onset 43 years	P079
***SETX***	**3**	c.934A>G	p.Ile312Val	0%-0%-0%			Spinal ALS, onset 67 years	P045
***SETX***	**3**	c.2344G>T	p.Val782Leu	0%-0%-0%		(**AR**) *ALS2:* c.37G > A (p.Gly13Arg) het [rs367871772, MAF *: 0%-0.01%-0.01%] (Class 4);	Lower motor neuron involvement, onset 21 years	P071
***SETX***	**3**	c.3494C>G	p.Ser1165Cys	0%-0%-0%			Spinal ALS, onset 72 years	P029
***SETX***	**3**	c.4220A>G	p.Asn1407Ser	0%-0%-0%	rs747050949		Bulbar ALS, onset 79 years	P069
***SETX***	**3**	c.4957A>G	p.Lys1653Glu	0%-0%-0%			Spinal ALS, onset 66 years	P088
***ANG***	**3**	c.61C>T	p.Pro21Ser	0%-0.02%-0.02%	rs149672657	(**HMN**) *TRPV4:* c.1006C > T (p.Arg336Cys) het [rs781229110, MAF *: 0%-0%-0%] (Class 3); (**Class 2**) *SQSTM1*: c.712A > G (p.Lys238Glu) het [rs11548633, MAF *: 0.24%-0.26%-0.24%] (**Class 2**) *SETX*: c.3229G > A (p.Asp1077Asn) het [rs145097270, MAF *: 0.08%-0.07%-0.11%]	Spinal ALS	P094
***CHMP2B***	**3**	c.36T>G	p.Asp12Glu	0%-0%-0%		(**Class 2**) *GRN*: c.264+7G > A (*p* = ?)het [rs60100877, MAF *: 0.5%-0.6%-0.53%]	Spinal ALS, onset 71 years	P074
***ERBB4***	**3**	c.421 + 5G>A	p.? HSF: Alteration of the WT donor site, most probably affecting splicing	0%-0%-0%	rs778195807		-	P024
***NRBB4***	**3**	c.1441A>G	p.Ile481Val	0%-0.01%-0%	rs368860175		Bulbar ALS, onset 70. One affected sibling	P092
***MATR3***	**3**	c.1132G>A	p.Ala378Thr	0%-0.03%-0.01%	rs201075828		Spinal ALS, onset 37 years	P099
***GARS1***	**3**	c.302G>A	p.Arg101His	0.02%-0.02%-0.04%	rs200887429		-	P005
***GARS1***	**3**	c.302G>A	p.Arg101His	0.02%-0.02%-0.04%	rs200887429		Spinal ALS	P030
***GARS1***	**3**	c.571A>T	p.Thr191Ser	0%-0%-0%	rs760133861	(**Class 2**) *GARS1*: c.803C > T (p.Thr268Ile) het [rs2230310, MAF *: 0.12%-0.48% 0.32%	Spinal ALS	P062
***GARS1***	**3**	c.1159G>A	p.Ala387Thr	0%-0%-0%	rs776528885		Bulbar ALS and FTD	P065
***GRN***	**3**	c.-8 + 7G>C	p?	0%-0%-0%			Bulbar ALS	P054
***GRN***	**3**	c.329G>A	p.Arg110Gln	0.02%-0.01%-0.01%	rs375439809		Neuroacanthocytosis	P110
***GRN***	**3**	c.1528C>T	p.Arg510Cys	0%-0%-0%	rs747873577		Spinal ALS	P034
***GRN***	**3**	c.1691G>A	p.Arg564His	0%-0%-0%			Spinal ALS	P035
***MAPT***	**3**	c.64G>A	p.Asp22Asn	0%-0%-0%	rs745662662	(**AR**) *IGHMBP2*: c.2362C > T (p.Arg788Ter) het [rs199839840, MAF *: 0%-0.01%-0% (Class5)	Spinal ALS	P059
***MAPT***	**3**	c.319G>A	p.Gly107Ser	0%-0%-0%	rs769901930		Spinal ALS and mild cognitive impairment	P057
***TRPV4***	**3**	c.113A>G	p.Asn38Ser	0.02%-0%-0%	rs527355587	(**Class 2**) *TRPV4:* c.2518G > A (p.Glu840Lys) het [rs55728855, MAF *: 0.24%-0.74%-0.63%	ALS monomelic type	P033
***TRPV4***	**3**	c.1496C>T	p.Pro499Leu	0.02%-0%-0%	rs115358347		-	P093

* MAF% (minor allele frequency) in 1000Genomes-Go-ESP–ExAC. Hem: hemizygous; het: heterozygous; HSF: Human Splicing Finder; (Suscep.): variants in susceptibility genes; (AR): variants in autosomal recessive genes; (HMN): variants in motor neuronopathy disease genes. het: heterozygous; (AR): variants in autosomal recessive genes.

## Supplementary Materials

The following are available online at https://www.mdpi.com/2077-0383/9/2/412/s1, Table S1: Reference sequences for transcript (NM) and protein (NP) of genes analyzed in the study; Table S2: Criteria applied in the classification of variants identified in ALS genes; Table S3: Gene variants in ALS susceptibility genes; Table S4: Heterozygous gene variants in autosomal recessive ALS genes; Table S5: Class-2 gene variants.
