# Supplementary material for: Sorting Rare ALS Genetic Variants by Targeted Re-Sequencing Panel in Italian Patients: OPTN, VCP, and SQSTM1 Variants Account for 3% of Rare Genetic Forms
Source: J Clin Med. 2020 Feb 3;9(2):412. doi: 10.3390/jcm9020412 (PMC7073901; doi:10.3390/jcm9020412)
Supplement: Supplementary file 1 [file jcm-09-00412-s001.pdf]

Supplementary Table S1. Reference sequences for transcript (NM) and protein (NP) of genes analyzed.

Gene	RefSeq mRNAs	RefSeq proteins
ALS2	NM_020919.3	NP_065970.2
ANG	NM_001145.4	NP_001136.1
APEX1	NM_001244249	NP_001231178
ASAH1	NM_004315.4	NP_004306.3
ATXN2	NM_002973.3	NP_002964.3
BSCL2	NM_001122955.3	NP_001116427
CHCHD10	NM_213720	NP_998885
CHMP2B	NM_014043.3	NP_054762.2
CRYM	NM_001888.3	NP_001879.1
CYP27A1	NM_000784.3	NP_000775.1
DAO	NM_001917.4	NP_001908.3
DCTN1	NM_004082.4	NP_004073.2
DPP6	NM_001039350.1_dupl14.1	NP_001034439.1_dupl14.1
ELP3	NM_018091.5	NP_060561.3
EPHA4	NM_004438.3	NP_004429.1
ERBB4	NM_005235.2	NP_005226.1
FIG4	NM_014845.5	NP_055660.1
FUS	NM_004960.3	NP_004951.1
GARS1	NM_002047.2	NP_002038.2
GRN	NM_002087.2	NP_002078.1
HNRNPA1	NM_002136	NP_002127
HNRNPA2B1	NM_031243.2	NP_112533
HNRNPA3	NM_194247.2	NP_919223.1
IGHMBP2	NM_002180.2	NP_002171.2
MAPT	NM_001123066.3	NP_001116538.2
MATR3	NM_199189.2	NP_954659.1
NEFH	NM_021076.3	NP_066554.2
NEK1	NM_001199397.1	NP_001186326.1
OPTN	NM_021980.4	NP_068815.2
PFN1	NM_005022	NP_005013
PNPLA6	NM_001166111.1	NP_001159583.1
REEP1	NM_001164730.1	NP_001158202
SETX	NM_015046.5	NP_055861.3
SIGMAR1	NM_005866.2	NP_005857.1
SOD1	NM_000454.4	NP_000445.1
SPAST	NM_014946.3	NP_055761.2
SPG11	NM_025137.3	NP_079413.3
SQSTM1	NM_003900.4	NP_003891.1
TAF15	NM_139215.2	NP_631961.1
TARDBP	NM_007375.3	NP_031401.1
TREM2	NM_018965.3	NP_061838.1
TRPV4	NM_021625.4	NP_067638.3
TUBA4A	NM_001278552	NP_001265481
UBQLN2	NM_013444.3	NP_038472.2
VAPB	NM_004738.4	NP_004729.1
VCP	NM_007126.3	NP_009057.1

Supplementary Table S2. Criteria applied in the classification of variants identified in ALS genes.

Class	Pathogenicity	Criteria *
**5**	Clearly pathogenic	**1.** Variants reported in the literature as pathogenic with supporting evidence; multiple independent cases, pedigree segregation studies and/or functional analysis<br>AND<br>**2.** Phenotype and inheritance pattern in patient correlates with the gene<br>**OR**<br>**1.** Null variant (nonsense, frameshift, canonical ±1 or 2 splice sites, initiation codon, or out of frame exon deletion) in a gene where loss of function is a known mechanism of disease
**4**	Likely to be pathogenic	**1.** Not described in the literature, or weak evidence for pathogenicity in published literature; no segregation or functional analysis available<br>AND<br>**2.** Phenotype and inheritance pattern in patient correlates with the gene<br>AND<br>**3.** Variants not included in Class5 (Missense/synonymous/intronic) for which location in gene and pathogenic mechanism are compatible with previously described pathogenic variants in the gene<br>AND<br>**4.** Multiple lines of computational evidence support a deleterious effect on the gene or gene product (conservation, evolutionary, splicing impact, etc.):<br>a) Missense variant: conserved amino acid, Polyphen 2 (HumVar) and SIFT concur in predicting deleterious effect or at least three splice prediction tools support an effect on splicing<br>b) Synonymous or intronic variants: nucleotide highly conserved across multiple species or three or more splice prediction tools support an effect on slicing or regulatory mechanism
**3**	Unknown significance (VUS)	**1.** Other criteria described for class 5, 4, 2 and 1 are not met<br>**OR**<br>1. The criteria for benign and pathogenic are contradictory
**2**	Unlikely to be pathogenic	**1.** Allele frequency is greater than expected for the prevalence disorder (>0.02% in the majority of the databases) or observation in controls incompatible with disease penetrance or pattern of inheritance<br>**OR**<br>**1.** Well-established in vitro or in vivo functional studies show no damaging effect on protein function or splicing<br>**OR**<br>**1.** A synonymous and intronic variants for which three or more splicing prediction algorithms predict no impact to the splice consensus sequence nor the creation of a new splice site AND the nucleotide is not highly conserved
**1**	Clearly not pathogenic	**1.** Frequency >1 % in dbSNP or in Exome Variant Server or ExAC

* Modified from Antoniadi et al. (2015)

Supplementary Table S3. Gene variants in ALS susceptibility genes.

Gene	Variant DNA	Variant protein	MAF*	DbSNP	N. of patient	Patient	Other variants
ATXN2	c.1472A>G	p.Asn491Ser	0.1% - 0.15% - 0.09%	rs117851901	2	P011<br>P081	-<br>-
ATXN2	c.1955A>G	p.His652Arg	0% - 0% - 0%		1	P108	-
ATXN2	c.2294A>G	p.Asn765Ser	0% - 0% - 0.02%	rs765735723	1	P091	**(AR)** *SETX*: c.2479A>G (p.Lys827Glu) het [rs150532677, MAF*: 0.02% - 0.07% - 0.1%](Class2);
ATXN2	c.2624A>G	p.Lys875Arg	0% - 0% - 0%		1	P085	**(Class 2)** *ERBB4*: c.882A>G (p.=) het [rs77309171, MAF*: 0.2% - 0.36% - 0.42%] (Class2); *VAPB*: c.390T>G (p.Asp130Glu) het [rs146459055, MAF*: 0.1% - 0.07% - 0.14%] (Class2);
ATXN2	c.2836C>T	p.Pro946Ser	0% - 0% - 0%	rs771952619	1	P080	**(Suscep.)** *ATXN2*: c.3000A>G (p.=) het [rs140262591, MAF*: 0.2% - 0.31% - 0.3%]; *HNRNPA3*: c.625T>C (p.Ser209Pro) het [MAF*: 0% - 0% - 0%]; **(Class 2)** *GRN*: c.264+7G>A het [rs60100877, MAF*: 0.5% - 0.6% - 0.53%];
ATXN2	c.3000A>G	p.=	0.2% - 0.31% - 0.3%	rs140262591	7	P075<br>P080<br>P014<br>P020<br>P032<br>P058<br>P090	**(ALS genes)** OPTN: c.910C>T (p.Leu304Phe) het [MAF*: 0% - 0% - 0%](Class3);<br>**(Suscep.)** *ATXN2*: c.2836C>T (p.Pro946Ser) het [rs771952619, MAF*: 0% - 0% - 0%]; *HNRNPA3*: c.625T>C (p.Ser209Pro) het [MAF*: 0% - 0% - 0%]; **(Class 2)** *GRN*: c.264+7G>A het [rs60100877, MAF*: 0.5% - 0.6% - 0.53%];<br>-<br>-<br>-<br>-<br>-
ATXN2	c.3322C>T	p.Pro1108Ser	0.04% - 0.11% - 0.07%	rs140242317	1	P112	**(ALS genes)** *VCP*: c.179A>G (p.Lys60Arg) het [MAF*: 0% - 0% - 0%] (Class3);
CRYM	c.47A>C	p.His16Pro	0% - 0% - 0%		2	P016<br>P019	**(Class 2)** *SETX*: c.472T>G (p.Leu158Val) het [rs145438764, MAF*: 0.16% - 0.4% - 0.37%] (Class2); *SQSTM1*: c.712A>G (p.Lys238Glu) het [rs11548633, MAF*: 0.24% - 0.26% - 0.24%] (Class2);<br>-
CYP27A1	c.256G>A	p.Val86Met	0.26% - 0.01% - 0.19%	rs200604732	1	P089	**(AR)** *IGHMBP2*: c.2837G>A (p.Arg946Gln) het [rs149824485, MAF*: 0% - 0.08% - 0.07%] (Class3);
CYP27A1	c.524C>T	p.Thr175Met	0.56% - 0.58% - 0.17%	rs2229381	1	P114	
CYP27A1	c.536A>G	p.Asn179Ser	0% - 0.02% - 0.01%	rs145080072	1	P037	
CYP27A1	c.827A>C	p.Asn276Thr	0% - 0% - 0%		1	P013	**(Class 2)** *GRN*: c.264+7G>A het [rs60100877, MAF*: 0.5% - 0.6% - 0.53%] (Class2);
DAO	c.627G>A	p.Trp209Ter	0% - 0% - 0%	rs766258671	1	P002	**(ALS genes)** *SQSTM1*: c.695C>T (p.Pro232Leu) het [rs757778292, MAF*: 0% - 0% - 0%](Class4); **(Suscep.)** *DCTN1*: c.652G>A (p.Glu218Lys) het [MAF*: 0% - 0% - 0%];

Gene	Variant DNA	Variant protein	MAF*	DbSNP	N. of patient	Patient	Other variants
DCTN1	c.586A>G	p.Ile196Val	0.32% - 0.55% - 0.8%	rs55862001	1	P028	-
DCTN1	c.652G>A	p.Glu218Lys	0% - 0% - 0%		1	P002	**(ALS genes)** *SQSTM1*: c.695C>T (p.Pro232Leu) het [rs757778292, MAF*: 0% - 0% - 0%](Class4); **(Suscep.)** *DAO*: c.627G>A (p.Trp209Ter) het [rs766258671, MAF*: 0% - 0% - 0%]
DCTN1	c.1208A>G	p.Glu403Gly	0% - 0% - 0%		1	P113	**(Class 2)** *SPG11*: c.258-6delT het [rs373234269; rs556942992, MAF*: 0.86% - 0.7% - 0.19%] (Class2); *SPG11*: c.4687A>G (p.Arg1563Gly) het [rs75430389, MAF*: 0.7% - 0.55% - 0.17%] (Class2);
DCTN1	c.1955T>G	p.Phe652Cys	0% - 0% - 0%		1	P012	**(Class 2)** *VAPB*: c.390T>G (p.Asp130Glu) het [rs146459055, MAF*: 0.1% - 0.07% - 0.14%] (Class2);
DCTN1	c.2185-6C>T	p.?	0% - 0% - 0%		1	P044	**(AR)** *IGHMBP2*: c.2043G>T (p.Glu681Asp) het [MAF*: 0% - 0% - 0%] (Class3);
DCTN1	c.2633A>G	p.Tyr878Cys	0% - 0% - 0%	rs778201974	1	P098	-
DCTN1	c.2657A>G	p.Tyr886Cys	0% - 0% - 0%	rs370085138	1	P048	-
DCTN1	c.3529+5G>A	p.?	0.26% - 0.69% - 0.59%	rs72466494	3	P067<br>P104<br>P049	-<br>-<br>-
DPP6	c.1786G>A	p.Ala596Thr	0.1% - 0.19% - 0.15%	rs188276022	1	P082	-
ELP3	c.1111A>G	p.Met371Val	0% - 0% - 0%	rs199903018	1	P021	-
ELP3	c.1459C>T	p.Arg487Trp	0.06% - 0.05% - 0.07%	rs139093061	1	P0117	**(AR)** *SPG11*: c.5314C>T (p.Arg1772Cys) het [rs769001849, MAF*: 0% - 0% - 0%] (Class2); *SETX*: c.472T>G (p.Leu158Val) het [rs145438764, MAF*: 0.16% - 0.4% - 0.37%] (Class2);
EPHA4	c.1711C>T	p.Arg571Trp	0% - 0.02% - 0.01%	rs371177966	1	P023	
EPHA4	c.1946G>A	p.Cys649Tyr	0% - 0% - 0%		1	P076	**(Class 2)** *GRN*: c.264+7G>A het [rs60100877, MAF*: 0.5% - 0.6% - 0.53%] (Class2);
HNRNPA3	c.196-7T>G	p.?	0.3% - 0.57% - 0.52%	rs150012936	3	P052<br>P053<br>P078	
HNRNPA3	c.625T>C	p.Ser209Pro	0% - 0% - 0%		1	P080	**(Suscep.)** *ATXN2*: c.3000A>G (p.=) het [rs140262591, MAF*: 0.2% - 0.31% - 0.3%]; *ATXN2*: c.2624A>G (p.Lys875Arg) het [MAF*: 0% - 0% - 0%]; **(Class 2)** *GRN*: c.264+7G>A het [rs60100877, MAF*: 0.5% - 0.6% - 0.53%];
HNRNPA3	c.827A>G	p.Asn276Ser	0.04% - 0.03% - 0%	rs143598547	1	P084	**(AR)** *PNPLA6*: c.856C>T (p.Arg286Trp) het [MAF*: 0% - 0% - 0%] (Class3);
NEFH	c.1054C>A	p.Arg352Ser	0.08% - 0.35% - 0.26%	rs149955255	4	P077<br>P051<br>P055<br>P086	**(Class 2)** *ANG*: c.208A>G (p.Ile70Val) het [rs121909541, MAF*: 0.02% - 0.05% - 0.06%] (Class2);<br>-<br>-<br>-

Gene	Variant DNA	Variant protein	MAF*	DbSNP	N. of patient	Patient	Other variants
NEK1	c.1942A>G	p.Lys648Glu	0% - 0.01% - 0.01%	rs371562840	1	P095	-
TREM2	c.184C>T	p.Arg62Cys	0% - 0% - 0.01%	rs201258314	1	P070	**(Class 2)** *TRPV4*: c.387-6C>T het [rs775634013, MAF*: 0% - 0% - 0%] (Class2);
TREM2	c.469C>T	p.His157Tyr	0.28% - 0.02% - 0.36%	rs2234255	1	P063	

* MAF: Minor Allele frequency (%) in 1000Genomes-Go-ESP–AC databases. het: heterozygous; (ALS genes) Variants in ALS genes; (Suscep.): variants in susceptibility genes; (AR): variants in autosomal recessive genes.

Supplementary Table S4. Heterozygous gene variants in autosomal recessive ALS genes.

Gene	Class	Variant DNA	Variant protein	MAF*	dbSNP	Additional variants	Patient
ALS2	3	c.3015T>G	p.Asp1005Glu	0% - 0% - 0%			P042
ALS2	3	c.4226A>G	p.Gln1409Arg	0% - 0% - 0%			P107
ASAH1	5	c.108_114delCTTTGCT	p.Ser36ArgfsTer2	0.12% - 0.13% - 0.04%	rs548868946	**(Class 2)** TRPV4: c.2518G>A (p.Glu840Lys) het [rs55728855, MAF*: 0.24% - 0.74% - 0.63%]	P064
ASAH1	3	c.640G>A	p.Val214Ile	0.06% - 0.02% - 0.02%	rs151320126		P027
ASAH1	3	c.766A>C	p.Ile256Leu	0% - 0.01% - 0.01%	rs374187681		P066
IGHMBP2	5	c.653delC	p.Thr218MetfsTer15	0% - 0% - 0%			P109
IGHMBP2	3	c.736A>G	p.Ile246Val	0% - 0.01% - 0%	rs377678376	**(Class 2)** TRPV4: c.2248G>A (p.Val750Ile) het [rs148171058, MAF*: 0.08%-0.15%-0.08%]; **(Class2)** GARS:c.803C>T (p.Thr268Ile) het [rs2230310, MAF*: 0.12%-0.48%-0.32%]	P046
IGHMBP2	5	c.752T>G	p.Leu251Arg	0% - 0% - 0%			P003
IGHMBP2	4	c.815T>C	p.Leu272Pro	0% - 0% - 0%	rs773582904		P038
PNPLA6	3	c.1106G>A	p.Arg369Lys	0% - 0% - 0%			P026
PNPLA6	3	c.1225A>C	p.Thr409Pro	0% - 0% - 0%			P001
PNPLA6	3	c.1621C>T	p.Arg541Cys	0% - 0% - 0%	rs745665473		P118
PNPLA6	3	c.3676C>T	p.Pro1226Ser	0% - 0% - 0%		**(Class 2)** TRPV4: c.2518G>A (p.Glu840Lys) het [rs55728855, MAF*: 0.24% - 0.74% - 0.63%]	P102
PNPLA6	5	c.4075C>T	p.Arg1359Trp	0% - 0.02% - 0.01%	rs374434303		P018
SIGMAR1	4	c.553G>C	p.Ala185Pro	0% - 0% - 0%			P056
SPG11	3	c.604A>G	p.Met202Val	0.02% - 0% - 0%	rs201875705		P031
SPG11	3	c.980T>C	p.Leu327Pro	0% - 0% - 0%	rs201444186		P083
SPG11	3	c.3453+8A>T	p.?	0% - 0% - 0%			P068

* MAF: Minor Allele frequency (%) in 1000Genomes-Go-ESP–ExAC databases.

Supplementary Table S5. Class 2 gene variants

Gene	Class	Variant DNA	Variant protein	MAF*	dbSNP	Patient
ANG	2	c.208A>G	p.Ile70Val	0.02% - 0.05% - 0.06%	rs121909541	P004<br>P116
ERBB4	2	c.882A>G	p.=	0.2% - 0.36% - 0.42%	rs77309171	P039
GARS1	2	c.803C>T	p.Thr268Ile	0.12% - 0.48% - 0.32%	rs2230310	P009
IGHMBP2	2	c.1756+4C>T	p:?	0% - 0% - 0.02%	rs778913429	P036
SETX	2	c.59G>A	p.Arg20His	0.5% - 0.75% - 0.9%	rs79740039	P010<br>P041<br>P047<br>P050<br>P097
SETX	2	c.472T>G	p.Leu158Val	0.16% - 0.4% - 0.37%	rs145438764	P061<br>P111
SETX	2	c.4660T>G	p.Cys1554Gly	0.58% - 0.31% - 0.58%	rs112089123	P101<br>P115
SPAST	2	c.131C>T	p.Ser44Leu	0.12% - 0.65% - 0.54%	rs121908515	P096
SPG11	2	c.2656T>C	p.Tyr886His	0.4% - 0.38% - 0.14%	rs139687202	P060
TRPV4	2	c.2498A>G	p.Asn833Ser	0.04% - 0.27% - 0.31%	rs116035946	P040
VAPB	2	c.390T>G	p.Asp130Glu	0.1% - 0.07% - 0.14%	rs146459055	P006

* MAF: Minor Allele frequency (%) in 000Genomes-Go-ESP–ExAC databases.
